# Characterizing the Environmental Health Literacy and Sensemaking of Indoor Air Quality of Research Participants

**DOI:** 10.3390/ijerph19042227

**Published:** 2022-02-16

**Authors:** Kathryn S. Tomsho, Erin Polka, Stacey Chacker, David Queeley, Marty Alvarez, Madeleine K. Scammell, Karen M. Emmons, Rima E. Rudd, Gary Adamkiewicz

**Affiliations:** 1 Department of Environmental Health, Harvard T.H. Chan School of Public Health, Boston, MA 02215, USA; malvarez@hsph.harvard.edu (M.A.); gadamkie@hsph.harvard.edu (G.A.); 2 Department of Environmental Health, Boston University School of Public Health, Boston, MA 02118, USA; polkaen@bu.edu (E.P.); mls@bu.edu (M.K.S.); 3 Health Resources in Action, Boston, MA 02116, USA; schacker@hria.org; 4 Codman Square Neighborhood Development Corporation, Dorchester, MA 02124, USA; david@csndc.com; 5 Department of Social and Behavioral Sciences, Harvard T.H. Chan School of Public Health, Boston, MA 02115, USA; kemmons@hsph.harvard.edu (K.M.E.); rrudd@hsph.harvard.edu (R.E.R.)

**Keywords:** environmental health literacy, indoor air quality, sensemaking, environmental health communication

## Abstract

This study is based on in-depth semi-structured interviews with the participants of an indoor air quality monitoring study. The purpose of the interviews was to capture participants’ perceptions of indoor air quality and engage them in a discussion of those factors that influenced their behavior. Interview study participants (*n* = 20) noted the importance of family health concerns and their own sensory awareness of possible contaminants. They discussed their level of personal control over their home environment as well as their access to needed resources. This study is based on grounded theory and applies interpretivist epistemological methods. Study findings offer insights into how people perceive their home environment and what influences their decision making and action. Analyses indicate that perceived agency, risk perception, access to resources, and information all influenced participants’ sense of ability to take action as well as their interest in taking action. These insights serve to challenge some of the current work in environmental health literacy which tends to focus on and measure an individual’s knowledge or skills. Our analysis suggests that consideration be given to a number of factors that include perceived agency, access to resources, and the quality of information provided.

## 1. Introduction

Providing accessible information regarding environmental exposures can facilitate practical outcomes, such as adopting exposure-reduction behaviors. It can also be a means of power sharing and bidirectional decision making in community-engaged research [1]. As a result, there is growing interest in identifying the best means of providing environmental health information to lay audiences and the resultant impacts on knowledge gain and behavior adoption [2,3,4]. Interest in this area has led to the genesis of a new research domain: environmental health literacy (EHL) [2,5]. Much of the early work associated with EHL “focused primarily on elucidating the attributes of EHL and on the stages of becoming literate about environmental health concepts and issues” [1]. A great deal of the focus has been on content knowledge (typically via pre-post assessments), adopting exposure-reduction behaviors, or creating tools to evaluate baseline levels of EHL [2,6,7,8,9,10,11,12,13,14,15,16].

Efforts to characterize EHL have generally assessed the impact on individuals or communities who have received environmental health information [2]. Characterizing contextual factors that influence the understanding of environmental hazards and engagement in health-protective behaviors can contribute to more effective and accessible environmental health interventions. It may also afford a holistic depiction of the range of factors that influence environmental health literacy for a given environmental hazard. 

This study is based on in-depth interviews with a sample of participants who had been involved in a research study measuring air quality within their homes. We explore how participants consider issues of indoor air quality in their home and what contextual factors they identify as they discuss decision making and action. Grounded theory affords the opportunity to explore emergent factors and their relationship to awareness, risk perception, and health-relevant behaviors.

The purpose of this analysis was to characterize the ways in which participants make sense of indoor air quality (IAQ) and how they respond or take action based on that sensemaking. Sensemaking refers to the process through which people interpret and understand phenomena. This can include rationales as to what is causing the phenomenon as well as actions and reactions to it. Sensemaking of IAQ was defined as the wide range of considerations and variables that contribute to how a person gives meaning to and internalizes the topic of air quality in their indoor home environment. This characterization can provide critical insight for future efforts to communicate with community members or groups who are affected by poor indoor air quality or have an interest in improving their indoor air quality.

### Study Context: Dorchester, Massachusetts

This effort focused on participants of an urban cohort who took part in an indoor air quality study in Dorchester, Massachusetts. All 78 participants hosted monitoring equipment in their homes and were provided the option of receiving their personal monitoring data back via a written report and meetings with the research team [16,17]. 

Dorchester, Massachusetts is a neighborhood in the southern part of Boston. In 2015, Dorchester comprised about 20% of Boston’s population [18]. The Dorchester population is diverse, with about 34% being foreign-born (as compared to 27% of Boston) and 45% identifying as Black/African American, 18% as Hispanic, 9% as Asian/Pacific Islander, 6% as other, and 22% as White in 2017 [19]. There is also a wide variety of levels of educational attainment, with 20% reporting less than a high school degree, 55% with a high school degree or some college, and 26% with a bachelor’s degree or higher in 2017 [19]. The housing infrastructure, with more multi-unit housing in Dorchester, also differs from that of Boston [19]. 

Dorchester has elevated rates of asthma, with 18% of adults reporting having asthma, compared to 12% of adults in Boston overall [20]. Dorchester also has higher rates of asthma emergency department visits among children age 3–5 years, 405.2 per 10,000 children, as compared to 285.4 per 10,000 children in Boston overall [20]. Asthma hospitalization rates are higher for Black and Latino residents, about 4 and 3 times the rate for White residents, respectively [20]. Asthma rates are also higher for Boston-area residents with lower income. Households making less than $25,000 per year were more likely to have chronic asthma as compared to those with a household income of $50,000 or more per year. 

The demographic and environmental characteristics of Dorchester lead to increased risks for poor air quality among its residents and thus has led to an interest in understanding indoor air quality within Dorchester households [20,21,22]. We engaged residents in the study of indoor air quality and then shared results with participants with the intention of providing them with information to inform action. 

## 2. Materials and Methods

### 2.1. Data Collection

Study Population: Participants for this formative research were selected from the Home-based Observation and Monitoring Exposure (HOME) Study which was conducted in Dorchester, Massachusetts between 2017 and 2019. The HOME Study, a project within the Center for Research on Environmental and Social Stressors in Housing Across the Life Course (CRESSH), measured a series of air pollution variables inside and outside participants’ homes. HOME study participants hosted an Environmental Multi-pollutant Monitoring Assembly (EMMA) device inside their homes for two weeks, one during a cold season and one during a warm season. The EMMA devices, described elsewhere, recorded in-home concentrations of PM_2.5_ and NO_2_, carbon monoxide, carbon dioxide, nitric oxide, nitrogen dioxide, temperature, relative humidity, and noise [16]. 

### 2.2. Study Population

All HOME participants who completed both seasons of monitoring were selected for our evaluation based on their categorical responses to the baseline question on the perception of Dorchester air quality. Possible responses to the perception of Dorchester air quality were: ‘Very bad’, ‘Bad’, ‘Good’, ‘Very good’, ‘I have never thought about it’, and ‘I am uncertain/don’t know’. This question was considered to be a proxy for prior consideration of air quality, in general. Participants were randomly sampled from each of the six response options for this question (please see Table S3 in Supplementary Materials for details regarding response rates for each survey response category).

Of the 78 HOME participants in Dorchester, 16 did not complete the second season of indoor air quality monitoring. The remaining 62 participants were classified based on their categorical responses to the baseline question on the perception of Dorchester air quality. Participants were randomly sampled from each of the categorical response bins for this question. The distribution of the responses on the baseline surveys for each response option was as follows: ‘Very bad’ 8.3%, ‘Bad’ 16.6%, ‘Good’ 34.7%, ‘Very good’ 2.7%, ‘Never thought about it’ 20.8%, and ‘Uncertain’ 16.7% of all participants. 

To match the distribution of responses in the baseline data, we aimed to interview participants in a manner that mirrored the original distribution of views on air quality in Dorchester with the 20 interviewed participants. 

### 2.3. Baseline Survey Data

Baseline survey data were collected for each participant during the initial home visit. A series of questions regarding perceptions, home characteristics, and home-health behaviors were posed to participants, and answers were collected via an oral survey. The semi-structured interview script can be found in Table S1 in the Supplementary Materials.

#### Qualitative Methods

We used the integration approach in which, “the quantitative methods are used to embellish a primarily qualitative study” [23]. Information from the quantitative component informed discussions and questions asked during the interviews. Baseline survey questions were also used to inform the sampling process. From the HOME study, 20 participants, selected as described above, were invited to participate in short (about 1 h) interviews with the first author. 

Interviews: Interviews were conducted in person for the first ten participants, and due to the onset of the COVID-19 pandemic, over the phone for the final ten participants. All interviews were audio-recorded with the consent of the participants. Audio recording failed for the first two participants interviewed over the phone. For these two interviews, the interviewer wrote detailed notes about the conversation. This information was ultimately used to help inform the creation of the data report-back materials but could not be included in the qualitative data analysis. Interviews were transcribed within 48 h of each interview by the interviewer, and detailed memos regarding the interviews were created. We obtained approval for these interviews and all associated materials from Harvard T.H. Chan School of Public Health’s Institutional Review Board (IRB 15-1756).

Participants were asked a series of questions about their in-home products and behaviors that could impact indoor concentrations of NO_2_ or PM_2.5_. Questions were open-ended and asked participants to describe whether they have candles, incense, air purifiers, or humidifiers in the home, and about their typical behaviors with those products in their home. Participants were also asked about their typical kitchen behaviors, their perceptions of their indoor air quality, and what concerns they had, if any, about their air quality. They were also asked what would inspire them to attend a report-back meeting, what their preferred method of receiving information back would be, and what made medical or scientific materials challenging or approachable for them. All interviews were completed before report backs were sent to participants. 

### 2.4. Health Literacy Assessment

Participants were also asked four questions from the BRIEF health literacy assessment, which asked participants to indicate their responses to the following questions using a 5-point Likert-type scale (ranges either always to never, or not at all to extremely):

1. How often do you have someone help you read medical materials?
2. How confident are you in filling out medical forms by yourself?
3. How often do you have problems learning about a medical condition because of difficulty understanding written information?
4. How often do you have a problem understanding what is told to you about your medical condition?

The score for the BRIEF assessments was summed for each participant (possible range for the score was from 4 to 20) [24]. Participants were then categorized based on their summed BRIEF assessment score. Scores were categorized as follows: Inadequate (4–12), marginal (13–16), and adequate (17–20) [24]. 

### 2.5. Data Analysis

***Epistemology*:** The research team took an interpretivist epistemological stance for this work [25,26]. In this approach, the researchers aim to characterize and understand a phenomenon by evaluating the reality of that phenomenon as experienced by individuals [26]. Through this work, we attempt to better understand the way in which individuals currently perceive their indoor home environment, specifically as it pertains to the creation and avoidance of indoor air pollutants. We additionally aim to better understand the way that participants attend to and contextualize indoor air quality (IAQ) information within their own homes. Grounded theory is inductive and is typically implemented to create or explore theory [27]. The novelty of environmental health literacy research makes grounded theory the most appropriate for this scenario in that it allows themes and categories within the data to emerge [28]. 

***Analysis*:** All transcripts were imported into NVivo 12 Pro. All coding and thematic analysis were completed within NVivo 12 Pro. The coding approach followed the three steps of grounded theory; open, axial, and selective coding [28]. Open coding was implemented first to generate categories of information found within the interview data [28]. This took a thematic approach, rather than a line-by-line analysis. Subsequently, axial coding was used to create links and thematic groupings of the codes identified through the open coding [28]. This identified relationships among themes, and identified core categories from which sub-categories stem [28]. We implemented Strauss and Corbin’s approach of categorizing the data along the dimensions of causal conditions, central/core phenomenon, contextual conditions, intervening conditions, action/interaction strategies, and consequences [29]. Finally, selective/integration coding was implemented to finalize and integrate the theory that emerged from the data [28]. This process ultimately created a visual model of the axial relationships identified in the axial coding and solidified the relationships generated by the analysis [28].

We examined the confirmability, transferability, and credibility [26,30] of our data as follows:

Confirmability refers to the “degree to which the research findings can be confirmed or corroborated by others” [26]. To achieve confirmability, it is suggested that research approaches include a confirmability audit or a trail of approaches and data sources [30]. Therefore, to establish confirmability, the research team kept a detailed outline of the research steps taken throughout the research project from initiation to the ultimate discussion of findings [30]. 

Transferability is a concept broadly akin to external validity in quantitative research [30]. However, rather than implementing randomization techniques to ensure generalizability, the team engaged in “thick description” to provide substantial contextual descriptions of the contexts in which the qualitative data were collected [30]. This informs future researchers of the context in which the data were produced and allows them to decide the utility of the methods and approach to produce those data for their own purposes [30].

Credibility can be determined via a variety of approaches, including extensive engagement, member-checking, and triangulation [30]. Due to the limited timeframe and resources, extensive engagement was not possible, as we used single interviews with participants. Instead, themes that emerged were presented to members of a locally based community organization for their input to determine whether our conclusions matched their interpretation and understanding of the local context. 

## 3. Results

The overall representativeness of the interviewed population as compared to the full Dorchester HOME Study population is summarized in Table 1. The subset of participants interviewed for the formative research was not statistically different from the other Dorchester HOME Study participants in terms of ethnicity, race, household income, or educational attainment. 

### 3.1. Open & Axial Coding

Sixteen categories emerged from the interview data, including the core category *Sensemaking of Indoor Air Quality (IAQ)*. *Sensemaking of IAQ* emerged as the core category by meeting the six requisite criteria outlined by Strauss & Corbin: (1) all categories relate to the core category; (2) the core category appears frequently; (3) data are not forced to relate to the core category; (4) the core category is sufficiently abstract to be used in alternative areas; (5) the theory grows and deepens its explanatory power throughout theory refinement; and (6) the core categorical concept stands in varying settings [28]. The core category and other subcategories are displayed along with their properties in Table S2, which also provides dimensionalized examples of each of the categories to demonstrate the variety within each of the categories [28].

### 3.2. Sensemaking of IAQ

Sensemaking of IAQ was described by participants on different scales (recent and past life experiences) and in different capacities (knowledge-based, emotion-based, and sensory-based).

**Figure 1:**
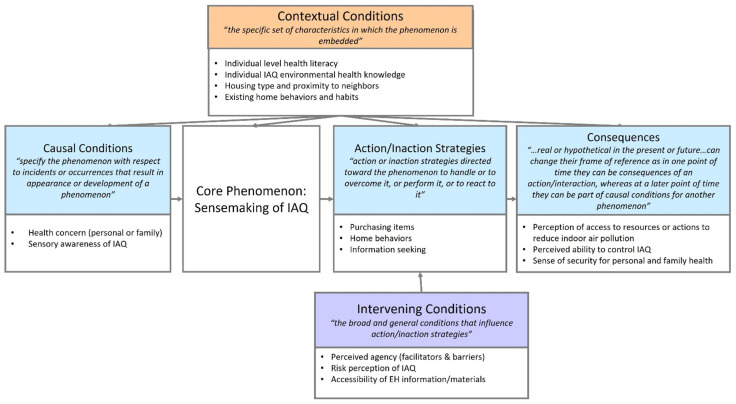
Axial coding relationships of grounded theory themes for Sensemaking of Indoor Air Quality (all category definitions are from Vollstedt and Rezat, 2019) [32].

The relationships of the codes identified in Table S2 are demonstrated via axial coding in Figure 1.

### 3.3. Causal Conditions 

Strauss and Corbin define causal conditions as those that “…specify the phenomenon with respect to incidents or occurrences that result in appearance or development of a phenomenon” [32]. Among participants, two themes were identified as being an impetus to substantial consideration or sensemaking of indoor air quality: (1) having a health concern for themselves or a household member; and (2) becoming aware or concerned about indoor air quality because of a sensory experience. 

***Concern for health*** was a primary motivator for participants to make sense of indoor air quality. This included general concern for maintaining the health of household members as well as avoiding potential triggers for acute health conditions. Asthma and other respiratory conditions were the main acute health concerns cited by participants:

Participant 18: “I feel that in my home the air quality for me is not good ‘cuz I was getting asthma attacks all the time, and I’ve got breathing problems sometimes. And I think my home—it’s still not that healthy for me. So that’s why…it’s a personal thing for me”.

Participants who mentioned asthma or respiratory conditions described a specific interest in understanding their home environment’s indoor air quality, and active information seeking regarding how to exert control over their IAQ. 

***Sensory awareness of IAQ*** was a second motivator for participants to spend time considering and seeking information about IAQ. Participants described having concerns about their IAQ because they noticed visual staining on their walls above heaters/fireplaces, mold in their home, or unwelcome scents such as from traffic or neighbors’ activities (i.e., smoking or cooking):

Participant 8: “My sister smokes weed when she comes to visit, and sometimes in the wintertime—when I can’t breathe with that, I have oil heat—I clean out the air…I have to get the furnace cleaned all the time. I can smell it—I think I can at least.”

Both sensory awareness and health concerns were driving factors for participants to engage in the sensemaking of IAQ. Participants who described either of these conditions described efforts to characterize the possible impact of indoor air pollutants within their space and to understand what abilities they have to improve their IAQ. 

### 3.4. Contextual Conditions

Contextual conditions are “…the specific set of characteristics in which the phenomenon is embedded…{which} also characterizes the special set of conditions in which action/interaction strategies take place to overcome, handle, or react to a certain phenomenon” [32]. Four contextual conditions influenced participants’ process of IAQ sensemaking, and their action/inaction strategies [32]. These were ‘individual health literacy’, ‘individual IAQ environmental health literacy’, ‘housing type and proximity to neighbors’, and ‘existing home behaviors and habits. 

***Individual health literacy*** scores, as measured by the BRIEF assessment of health literacy, ranged from marginal to adequate among interviewed participants, with no participants in the inadequate health literacy category. Participants described factors of medical communications that impacted their ability to contextualize and act on medical or scientific information. Namely, jargon, complex sentence structure, and rapid oral speech were the primary hindrances described: 

Participant 13: “People are … it seems like the sentence structure is different. It’s like—people don’t construct sentences in the way that they used to … that makes things plain. And got the point across… now it seems like people just want to go on and on and on with a bunch of words. And then at the very—and you gotta pick out what the important stuff really is, instead of just saying it. And it’s—it’s aggravating. It’s like come on, can you get to the point?… Sometimes it’s the words that they’re using and all that—but most times I think they just—I don’t know it seems like they just want to go on and on…”

Jargon and complex or dense written information led to confusion as to what the main messages of the communications were and muddled the participants’ understanding of what they needed to remember and what actions they could take to protect their personal health. 

***Individual IAQ environmental health knowledge*** referred to participants’ lived experiences that impacted their comfort, understanding, and use of IAQ environmental health information. This included topical knowledge about IAQ (such as sources, exposure routes, vulnerable or susceptible populations, and relevant health outcomes), as well as identification of possible actions that can address IAQ or relevant agencies. Individual IAQ environmental health knowledge varied greatly across the participants in terms of their awareness of the pollutants themselves and possible impacts. There were many participants who had never heard of either ‘nitrogen dioxide’ or ‘particulate matter’ who described concerns for vulnerable populations (for example in the context of the elevated rates of asthma in their community) and also described sources of each of the pollutants (such as smoking or cooking with the stove). One participant who did not recognize particulate matter or nitrogen dioxide described deep familiarity with actions she could take to improve her indoor air quality to protect her child with asthma:

Participant 4: “Nobody’s allowed to smoke in my house. I don’t use air fresheners –, I use fans, I don’t really use the air conditioner that much. {My daughter’s} not really allowed to have little stuffed animals…changing her bedding. She has—covers on her bed too– so for the dust mites and things like that. She has an air purifier in her room, I have one in my room…I’ve looked a lot online. Especially, the children’s hospital has like a whole section on air pollution and how to make your—your home more—I guess people who have asthma—to make it more safe. And definitely her doctors—just getting more information from them. Pamphlets from her school…” 

Despite not being familiar with the technical terms for particulate matter and nitrogen dioxide, some participants still described the perceived importance of IAQ and actions they took to address their home’s IAQ, as well as trusted sources of information for IAQ, such as doctors or hospital websites. 

Conversely, some participants who expressed familiarity with IAQ and technical language were generally unconcerned about their home’s IAQ:

Participant 3: “…it’s more the outside environment. I don’t think much about—I don’t think much about indoor air pollution. I don’t know that much about it. I probably know more about—or I’m more aware about outdoor air pollution… I don’t really think too much about indoor air pollution, so I’m really curious to see what the report might say.”

This range of awareness of indoor air pollution, comfort-seeking, and interpreting environmental information impacted the ways in which participants reacted to their perceptions of IAQ. However, the causal conditions (health concern or sensory awareness) continued to be the primary drivers for participants to take action to improve indoor air quality. 

***Housing type and proximity to neighbors*** were factors that participants described as impacting their IAQ or affecting their decision/ability to act to address their home’s IAQ. Specifically, participants who lived in multi-unit housing, and/or were renters, described themselves as having little control over their IAQ either because they were unable to address structural problems in their homes or because neighbors’ air quality impacted their own:

Participant 14: “Before I moved here, I owned the house—my home…so I kept my basement really clean and dry. Now I’m a renter, and the last two apartments I’ve had don’t have stove vents or any options like that.”

Participant 13: “It doesn’t matter what—it’s always cold in here—and you always smell somebody else’s apartment through the vent in the bathroom. It’s the vent in the bathroom the odors come through. It’s just—just not good…They’re all connected—the apartment—so I mean they don’t even—it’s pointless I don’t even know why they did that but, anyways, —if somebody next door is smoking cigarettes or smoking weed, you’re definitely going to smell it.”

In contrast, participants who were single-family homeowners expressed less frustration with their IAQ, and indicated that they either were unconcerned with it or felt as though they had the ability to change behaviors or infrastructure in their home if need be:

Participant 19: “Um—I guess it’s not up there on the list, to be honest…it’s maybe, if I had to rank them, maybe the 10th thing that I think about on a day-to-day basis {laughing}. I guess that I take it for granted, to be honest.”

Housing type and impact from neighbors on IAQ influenced participants’ awareness (in terms of having worried about it) and sense of control over their home’s IAQ. This, in turn, impacted the actions that they took (or did not take), and ultimately their sense of security over their home’s IAQ. 

Finally, ***existing home behaviors and habits*** described typical lifestyle patterns in terms relevant to IAQ such as candle/incense use, kitchen vent use, window opening behaviors, air purifier/humidifier use, and other relevant activities. Participants described a wide variety of typical home behaviors, including their routines for kitchen use, typical kitchen vent behaviors, and what motivated or prevented them from using candles, incense, or air purifiers and humidifiers.

### 3.5. Action/Inaction Strategies

Action and interaction strategies are those that impact the phenomenon of interest in terms of the individual’s reaction to respond or overcome it, and ultimately lead to the consequences of the phenomenon [32]. Three action/inaction strategies emerged from this analysis: (1) participants purchased items intended to address IAQ; (2) they chose to engage in behaviors in their home to address IAQ; and (3) they sought additional information about IAQ. 

***Purchasing items*** was one of the strategies employed by participants to control or change their indoor air quality. Multiple participants had purchased a humidifier and/or an air purifier to improve their home’s IAQ. Many of these participants did so in response to an acute health concern (such as asthma):

Participant 4: “{My daughter} has an air purifier in her room, I have one in my room…it was expensive, but it was worth it. Cuz my daughter doesn’t have as many flare-ups in the home as much as she used to.”

These purchases were made typically at the suggestion of a physician who indicated it may have a medical benefit. However, there were also participants who described purchasing air purifiers to improve their IAQ, despite being unsure of the true impact:

Participant 7: “I read online—so I had read that it helps with—breathing…but it could all be in my head cuz I swear since the ion cleaner, which is probably not cleaning anything—but I said the thing was almost like 200–300 dollars, so in my mind, I gotta pretend it’s doing something”

***Home behaviors*** taken by participants to address indoor air quality extended beyond purchasing items specifically intended to improve air quality. Participants also described actions they took in their homes to address IAQ that did not involve purchasing humidifiers or air purifiers. These included actions such as purchasing or mixing cleaning solutions that were ‘green’ and certain window/door opening behaviors to improve air circulation:

Participant 16: I got a whole bunch of plants in the house…and also, the German practice of opening the windows for 15 min and letting the air go through the house.”

Other participants suggested that they preferred to keep their windows closed to protect their indoor air quality:

Participant 9:” I personally like to keep windows closed. Cuz I just like to keep one quality of air circulating through. Cuz I find that I notice a lot of times windows are open, I see a lot of dirt coming in. It’s probably also because of asthma and things—pollen and things like that. So—so for me, I think I tend to just do things that will circulate and clean the air.”

The desire to keep windows closed was typically related to a desire to keep pollen or ‘dirty air’ outside and clean air inside, or to avoid the loss of warmth inside in the winter. 

***Seeking information about IAQ*** was the final action/inaction strategy described by the participants. Those who had not specifically sought out information about IAQ described it as not feeling ‘relevant’ to their own lives. Those who had sought information did so after learning about a medical condition, or having concern from sensory perceptions that led them to believe they had poor air quality:

Participant 8: “…okay, this is in my house, where’d it come from! … Yea—I read—the um, books from the doctor’s office. And, sometimes I just look on the internet—like different stuff I read.”

Participants’ decisions to engage in certain action or inaction strategies (such as purchasing additional items, adjusting their in-home behaviors, or seeking additional information) were influenced by both contextual and causal conditions. 

### 3.6. Intervening Conditions

Intervening conditions refer to, “the broad and general conditions that influence action/inaction strategies” [32]. There were three mitigating factors that impact participants’ action or inaction strategies. These included the participants’ perceived agency to induce change or control their home’s indoor air quality, their risk perception of indoor air quality in their homes, and the accessibility of environmental health information. 

***Perceived agency*** was the most prevalent hindrance for participants describing their control over their home’s IAQ. Multiple participants described a desire to address their indoor air quality but feeling as though they were unable to do so either because they were renters, they could not afford the solutions that they desired, or they were in close proximity to others who they believed negatively affected the IAQ (such as neighbors or family members). Specifically, the cost of air purifiers was prohibitive to some participants:

Participant 13: “I would just like to… I mean I know there’s nothing that can be done or nothing that will be done, but …I just wish that…I had cleaner air to breathe. That’s all. I wish I could afford air filters; you know.”

In addition to the cost burden of air purifiers and humidifiers, the cost of installing different types of kitchen vents was also cited as a barrier by participants. Among some participants who had humidifiers or purifiers, there was also skepticism as to whether they were actually effective, particularly for the amount of effort they required for upkeep:

Participant 9: “I used to have a humidifier, I just felt it was—cuz I bought the filter and everything, but the process of everything…you still seen the dark film. And that’s when I was like uhhh okay is this really working?”

Alternatively, some participants noted that knowing where to look for information (such as trusted family members or coworkers, or specific resources online) made them feel more confident and better able to control their IAQ. Additionally, participants who described having engaged in a project to address a different environmental concern in their home before (such as removing asbestos or lead paint) were less likely to express a sense of frustration or futility when considering the possibility of changing something in their homes in the future to address IAQ:

Participant 2: “…I tested the water lead levels after the stories were coming out about the Boston Public Schools—I tested the soil lead levels. We paint{ed} over some chipped paint. I sort of understood that indoor air quality was an emerging area of health concern, but I didn’t understand it very well…There are a big swatch of—am I okay I don’t need to worry or I’m not okay, and then I could start breaking it down. I’m like—oh—okay! I can run the fan when I’m cooking—it’s not just for smell. Maybe I’m making things a little healthier—I could do that.”

Conversely, some participants described specific barriers to being able to engage in the action/inaction strategies. These were largely due to not being able to make structural changes to their home because they are renters and/or their IAQ is impacted by their neighbors. Some participants also identified solutions to IAQ that were unaffordable (such as air purifiers) or having trouble finding accessible information on the topic to help inform their decision making. 

***Risk perception*** of IAQ varied widely among participants and was influenced by the two causal conditions, health concern and sensory awareness of IAQ. Some participants indicated that being involved in the study was the first time they had considered indoor air quality as a possible concern in their home:

Participant 1: “It seems like you’re reaching a niche that’s not really high on my awareness level. Well, I really don’t think of it being unhealthy in our home. And if the study says that there’s a big difference between households and certain things make a difference…then I’d like to know for my own—how I should…just something to let people know.”

While some participants expressed low concern over indoor air quality and the potential health impacts, other participants expressed intense concern for the possible health risks associated with IAQ:

Participant 18: “For me—it’s vital to my life. In terms of survival. As I said I have respiratory problems. It’s very important I have clean air. Clean environment. At all times.”

Participants’ risk perception of IAQ impacted their motivation whether to purchase items to remove indoor air pollutants, to alter their typical home behaviors and whether they had spent time actively seeking additional information about indoor air quality. 

***Accessibility of environmental health information*** was described by many participants as a challenge to understanding what steps to take:

Participant 20: “I think some scientists’ reports tend to be dense—and tend to think that big words make it sound important. And almost everything could be um … it’s not dumbed down, but put in more readable common language…and I think when you’re distributing information to the community, to include all of the technical information…but they could be really clear language…Sometimes it’s you know—long sentences with footnotes, and this that and the other thing. They’re very hard to follow.”

Participants consistently described a desire for more clarity in environmental health communications, regardless of their educational background. The most offered challenges were jargon, complex sentence structure, and ineffective translation of scientific concepts.

### 3.7. Consequences

Consequences are the general results of the action or inaction strategies to the phenomenon. The consequences can vary over time, as well as be hypothetical or real [32]. There were three consequences that resulted from the action/inaction strategies and intervening conditions related to the sensemaking of IAQ: (1) perception of access to resources or actions that can reduce indoor air pollution; (2) perceived ability to control IAQ; and (3) sense of security for personal and family health. 

Those who had experienced some sort of sensory indication of a possible IAQ problem and/or had a family member in the home with an acute health concern were more likely to engage in these action strategies. Those who had a low risk perception of indoor air quality were less likely to have someone in the home with a respiratory health condition or to have become aware of a specific air quality concern in their home. They were, in turn, less likely to have a concern about their ability to control IAQ or access resources to address IAQ. Additionally, they were less likely to express that they were concerned about the ability to maintain their own or their family’s health within their home.

Conversely, participants who had a family member with a specific health concern or had an acute awareness of an IAQ concern were those who reported seeking information and researching information about how to adjust their home behaviors specifically to address their IAQ. These efforts were often impacted by contextual conditions or intervening conditions that mitigated their abilities to engage successfully in the action/inaction strategies. Specifically, there were multiple barriers that participants described as being beyond their control (such as home structures or neighbors’ behaviors, or financial burdens) that hindered their ability to address IAQ concerns. This left those participants feeling frustrated and with a sense of futility about their prospects for taking control of their indoor air and health.

There was variability in the distribution of levels of the perceived risk of IAQ and perceived self-agency to control IAQ among participants, although none of the demographic variables were statistically significantly different (see Tables S4 and S5 in Supplementary Materials). Fifty-seven percent of participants who reported having little concern regarding their IAQ, or not perceiving it to be a risk for themselves were white. Sixty-four percent of participants reporting that they perceived IAQ to be very pertinent to their lives or carrying quite a bit of risk were Black or African American. Additionally, participants who reported having positive perceived self-agency to address their indoor air quality were primarily those with adequate health literacy (according to the BRIEF assessment of health literacy) [31]. Future efforts should explore the variability of demographics across these thematic constructs.

## 4. Discussion

Findings from this in-depth analysis of semi-structured interviews, building upon baseline data, serve to contextualize the factors that influence participants’ understanding of IAQ and their subsequent actions/inactions. Through this work, we identify one primary barrier to action to be the perception that participants are unable to achieve healthy IAQ because they cannot afford air purifiers or humidifiers. 

A second barrier to action participants described was access to trustworthy, accessible environmental health information. Participants described the challenges they had engaging with scientific materials because of jargon or complex sentence structures. 

It is evident from this work that there are factors beyond the individual’s literacy and educational background that influence their engagement with IAQ topics and their decisions or agency to address their IAQ. 

### Limitations

Although qualitative interviews can provide valuable insight regarding the ways in which people make sense of indoor air quality, this study has several limitations. First, this sample of HOME Study participants may not be representative of all study participants in terms of what impacts their sensemaking of IAQ. Though the sampling process intended to randomly sample participants from differing perspectives on outdoor quality in Dorchester, this may not be a suitable proxy to capture the full range of perspectives and themes relevant to the sensemaking of IAQ. The HOME Study participants may also not be representative of the general Dorchester, Massachusetts population. Those who participated in the HOME Study may have more awareness of air quality, in general, which led them to be interested in participating and learning more about their own indoor environment. Additionally, due to the loss of follow-up and non-response from participants, those who originally indicated they believed the air quality in Dorchester was ‘Good’ were underrepresented in the interviews, and those who believed that the air quality was ‘Very bad’ were overrepresented. 

The interview process spanned the two-week period during which the first Boston-area COVID-19 pandemic restrictions went into effect. This led to participants spending significantly more time at home and may have influenced their perceptions of indoor air quality and its heightened relevance to their personal health. 

The variation in educational attainment and health literacy levels of the participants of the interviews was also limited. Seventy percent (70%) of interview participants had a bachelor’s degree or higher, leaving only 30% of participants with up to some college education. Additionally, there were no participants who reported having inadequate health literacy, although nearly half of participants had marginal health literacy. The unevenness of representation across these variables may impact the suitability of this framework for other populations. The framework should be evaluated in additional populations with more variability across both health literacy scores and educational attainment.

The sensemaking of IAQ within the Dorchester community may also not be representative of other populations’ IAQ sensemaking. For example, during the time frame of this effort, there were substantial air quality concerns in the western portion of the United States from wildfires. While these were not front-of-mind for participants in Dorchester considering their indoor air quality, this may differ for communities who have been dramatically impacted by the air quality effects from fires. We believe that this framework will be suitable in different environments and populations but will need to be tested in a variety of additional contexts. 

## 5. Study Implications

Study findings contribute to theory and model building for environmental health literacy explorations *as well as* for concrete *actions* that may enhance future efforts to inform the public and enhance their ability to take needed action.

### 5.1. Importance of Contextual Issues

Within this effort, participants described awareness or concern regarding their IAQ and were often able to identify challenges (such as neighbors’ behaviors) and solutions (such as air purifiers or vents/fans) that they felt unable to access or address within their own home contexts. Additionally, participants referenced seeking environmental information—either written or oral—that they found to be inaccessible due to complex sentence structure or jargon. These findings may indicate challenges to the existing conceptualizations of environmental health literacy on two fronts: (1) that environmental health literacy is an attribute of an individual/community [1], rather than an interaction between environmental communicators and information seekers; and (2) that the direct connection between knowledge and enacting solutions is an appropriate metric for evaluating environmental health literacy [1,2]. This effort generates hypotheses to be evaluated in future work. Specifically:

1. Contextual barriers disrupt the proposed linear continuum between environmental (IAQ) knowledge and action.
  - Barriers to resources prevent participants from acting on IAQ information.
  - Cost barriers prevent participants from acting on IAQ information.

2. Environmental communications with high demand reduce readers’ motivation/impetus to act on them.
  - Environmental health materials with high demand leads to inaction.
  - Environmental health materials with high demand leads participants to have low perceived self-efficacy to take action.

To achieve ‘proficiency’ or advanced levels in existing conceptualizations of EHL, individuals are expected to be able to seek out, comprehend, and evaluate environmental health and science materials [1,2,33]. This necessitates interactions with materials created by environmental health professionals but fails to account for the accessibility of those materials in terms of literacy or numeracy demand. This work suggests expanding the model of environmental health literacy. The focus on the individual/community’s ability to navigate the information fails to consider a broader range of factors, including the messages they encounter [34]. If available environmental exposure/health communications are not accessible, then users will subsequently be unable to implement information within them. 

### 5.2. Expanding the Definition of Environmental Health Literacy

Definitions and suggested models for environmental health literacy continue to evolve. An early definition from the Society for Public Health Education focused on the skills and abilities of individuals:

Environmental health literacy integrates concepts from both environmental literacy and health literacy to develop the wide range of skills and competencies that people need in order to seek out, comprehend, evaluate, and use environmental health information to make informed choices, reduce health risks, improve quality of life, and protect the environment [1].

Finn and O’Fallon built upon this definition in their seminal 2017 paper describing the origins of EHL and the possibilities for future potential. Within that work, they further posited that “Individuals who are proficient in EHL are able to recognize their exposures and exert some manner of control over them rather than feeling as if ‘there’s nothing I can do.’” [1]. This moved the definition further by implicitly acknowledging the importance of perceived-self agency to act within the framework of EHL. It also suggested that EHL is not an all-encompassing attribute, but rather is specific to and may vary across different environmental hazards based on personal experiences [1].

The most recent definition was offered by Dr. Hoover in the first book on EHL:

…environmental health literacy can be defined as an emerging and evolving multidisciplinary field that seeks to better understand how individuals and communities make sense of and act on health-related information about environmental hazards. This definition makes clear that EHL requires basic scientific knowledge about contaminants, human health, and exposure pathways; however, such knowledge may not be sufficient for action. Rather, people at more advanced levels of EHL also need to understand the complicated roles, responsibilities, and uncertainties related to environmental health decisions and solution implementation processes [5].

Inherent in this definition is the importance of topical knowledge as well as ownership and empowerment regarding the decisions of how to act upon that knowledge. However, there are two important inclusions: first, that knowledge may not necessarily lead to exposure-reduction actions, and second, that there is a hierarchy of EHL levels, the top-most of which require a sophisticated understanding of stakeholders, scientific uncertainty, and the application of those knowledge components into their application towards solutions to reduce exposures. 

Together, these definitions indicate an expanding perspective on EHL from a sole focus on individual skills and abilities to apply environmental health knowledge to an acknowledgment of factors external to the individual (e.g., policies, systems, and environmental factors). 

The early definitions of EHL offered in the literature emphasize the importance of knowledge and capacity-building of individuals and communities to address or reduce relevant environmental exposures. Within these definitions, the onus of knowledge and action is within the individual or community experiencing exposure [1,2,5]. What we find to be lacking from each of these definitions is the identification and removal of barriers that exist between the implied continuum (or hierarchy) of environmental exposure, environmental contaminant knowledge, and the ability to access information and act upon it.

### 5.3. Supporting Action

The second implication of these early conceptualizations of EHL is that the ‘advanced level’ of EHL, or those who are environmentally-health-literate, are those who are able to implement solutions to reduce exposures, among other capabilities [1]. While there are some exposure scenarios in which there are widely accessible, low-cost solutions (such as opening windows to reduce indoor air pollution), there are exposure scenarios in which solutions may involve high cost or access to resources that are not widely accessible. Financial barriers may therefore pose a barrier to achieving EHL in its existing conceptualizations [1]. Focusing on individuals’ and communities’ ultimate ability to take ‘appropriate action’ to solve their exposure scenario, without acknowledging, measuring, and removing existing barriers, will characterize those who encounter barriers as having lower EHL, when in fact, they are possibly lacking access to the resources to remove barriers. ‘Raising EHL’ in these scenarios, without removing barriers, will be ineffective and will risk stigmatizing individuals and communities.

Adapting the definition of EHL has implications for appropriate measurements. If EHL is defined as an attribute or skillset of an individual navigating information and associated health behaviors, then measurement tools that assess individual knowledge and action are appropriate [35]. If, rather, EHL is a broader phenomenon that encapsulates individual skills and capacities in addition to environmental messages, existing behaviors, and structural factors, then measurements at the individual level will be insufficient. 

### 5.4. Informing Action

Message creators should additionally determine what potential barriers exist between the knowledge of exposure and the ‘appropriate’ action to reduce exposure. The message should assist in facilitating the removal of the barriers, rather than solely providing behavior suggestions for the reader. This can reduce the stigmatization of populations who have more barriers between knowledge and action. 

Additional efforts are needed to further develop our understanding of how people make sense of specific environmental health exposures. Findings from this study may provide insight for future inductive efforts to characterize populations’ environmental health literacy of specific exposures. 

## 6. Conclusions

This effort expanded beyond a measure of the skills and abilities of individuals to explore additional factors that facilitate or inhibit action to address environmental exposure. We found that participants who had family members with health conditions or could detect an IAQ concern via their senses were more likely to engage in the process of seeking information and behaviors that they could change to control their IAQ. We found that there were barriers that may have hindered their efforts and should be considered in future communication or intervention efforts.

## Figures and Tables

**Table 1:** Demographics of Interviewed Participants and Dorchester HOME Study Participants.

	Total	No Interview	Interview	*p*-Value
	(*N* = 78)	(*N* = 58)	(*N* = 20)	
Race				0.36
White	28 (35.9%)	20 (34.5%)	8 (40.0%)	
Asian	9 (11.5%)	8 (13.8%)	1 (5.0%)	
Black or African American	27 (34.6%)	18 (31.0%)	9 (45.0%)	
Other	10 (12.8%)	9 (15.5%)	1 (5.0%)	
Missing	4 (5.1%)	3 (5.2%)	1 (5.0%)	
Hispanic				0.26
No, Not Hispanic	66 (84.6%)	47 (81.0%)	19 (95.0%)	
Yes, Hispanic	12 (15.4%)	11 (19.0%)	1 (5.0%)	
Educational Attainment				0.7
Up to high school diploma, GED	14 (17.9%)	12 (20.7%)	2 (10.0%)	
Some college or associate degree	17 (21.8%)	13 (22.4%)	4 (20.0%)	
Bachelor’s degree	17 (21.8%)	11 (19.0%)	6 (30.0%)	
Post graduate degree	29 (37.2%)	21 (36.2%)	8 (40.0%)	
Refused to answer	1 (1.3%)	1 (1.7%)	0 (0%)	
Household Income				0.54
Less than $20,000	22 (28.2%)	19 (32.8%)	3 (15.0%)	
$20,000 to $50,000	14 (17.9%)	11 (19.0%)	3 (15.0%)	
$50,000 to $100,000	21 (26.9%)	13 (22.4%)	8 (40.0%)	
$100,000 or more	17 (21.8%)	12 (20.7%)	5 (25.0%)	
Don’t know	1 (1.3%)	1 (1.7%)	0 (0%)	
Refused to answer	3 (3.8%)	2 (3.4%)	1 (5.0%)	

Participants’ BRIEF assessment of health literacy scores ranged from marginal (*n* = 8) to adequate (*n* = 12) [31].

## Supplementary Materials

The following are available online at www.mdpi.com/article/10.3390/ijerph19042227/s1, Table S1: Semi Structured Interview Script, Table S2: Axial Coding Domains, Dimensions, and Examples for Sensemaking of IAQ, Table S3: Distribution of Baseline Survey Response on Dorchester Air Quality and Interview Sampling Response Rates, Table S4: Comparison of Demographics for Risk Perception Levels, Table S5: Comparison of Demographics for Perceived Self Agency Levels.
