# Supplementary material for: Characterizing the Environmental Health Literacy and Sensemaking of Indoor Air Quality of Research Participants
Source: Int J Environ Res Public Health. 2022 Feb 16;19(4):2227. doi: 10.3390/ijerph19042227 (PMC8871841; doi:10.3390/ijerph19042227)
Supplement: Supplementary file 1 [file ijerph-19-02227-s001.zip › ijerph-1514128-supplementary.pdf]

Supplementary Materials

Table S1: Semi Structured Interview Script

- We want to better understand how people think about different ideas. I'll say a word or topic, and then I would like you to list any words or phrases that come to your mind. This is very informal, so please feel free to list the first things that come to you.
- Prompt words:
  - Environmental Health
  - Air Pollution
    - Indoor Air Pollution
- The next section of questions is to help me learn more about peoples' lifestyles in their homes. There are no right or wrong answers, but please give as much detail as you can.
  - When I say the words 'air pollution', does anything come to mind?
    - Do you picture anything in your mind, like a certain place or a source of pollution?
  - Have you ever taken any actions or thought about taking actions in your home to make it 'healthy' in terms of air quality?
    - [If yes, what were they & what led you to do so?]
    - [If no, continue to next question]
  - What types of information, if any, have you looked for about how to make your home 'healthy'?
  - Have you, or anyone in your family, ever looked for information about your home's indoor air quality?
    - [If yes, what led you to do so?]
    - [If yes, are there specific tings in your home that you think impact your home's air quality?]

- This set of questions is about things you may use in your home, and choices you might make about your home. Again, there are no right or wrong answers, but the details you give me will be helpful.
  - How much time do you typically spend in the kitchen (this can include cooking with the stove, meal preparation, or eating)?
    - Is the time you spend in the kitchen different on weekdays vs. weekends?
      - [If yes, why?]
- When you cook with the oven or stove, do you typically use the stove vent or open a window?
    - What leads you to use [or not to use] the stove vent?
  - Do you tend to use the stove or oven more during any season or time of the year?
  - Do you use candles or incense in your home?
    - [If no, is there a reason?]
    - [If yes, are there certain times that you use them? Or anything that prompts you to use them?]
    - [If yes, what types of candles do you typically use?]
  - Do you have air purifiers in your home?
    - [If no, is there a reason?]
    - [If yes, are there certain times that you use them? Or anything that prompts you to use them?]
    - [If yes, what type of purifier do you use?]

• The next section of questions is to help our research team as we get ready to return our air quality monitoring results to the study participants. I'm going to ask a few questions about giving your data back, and what your preferences would be. This will be helpful for our team to make sure we give data back in a way that is useful for participants.<br><br>o Would you be interested in learning about the air quality measurements we took in your home?<br>▪ [If yes, what leads you to be interested]<br>▪ [If no, could you describe the reason it is not of interest to you? Or what could make it of interest?]<br>o Do you have a preference in receiving that information in a printed version, through attending a group meeting, or both?<br>o If we were to have a group meeting, is there a time of day/week that you would prefer? Such as a weeknight, weekend day, etc. …
We've come to the end of the questions that I've prepared for this interview. Is there anything else that you'd like me to know about your home & indoor home health? Is there anything else that you think I should know about your residence that may impact some of the descriptions/answers you provided?

Table S2: Axial Coding Domains, Dimensions, and Examples for Sensemaking of IAQ

Broad Axial Coding Category	Category or Subcategory	Properties or Dimensions	Dimensionalized Examples	
Causal Conditions	Health concern (personal or family member)	Spectrum of worry or focus on health status and possible impacts to family's health from air quality	General concern for personal or family members' health	Acute concern for personal or family members' health (such as a specific medical condition or vulnerable group)
	Sensory awareness of IAQ	Indication of awareness of indoor air quality via sight, smell, or taste	Awareness of air pollution generally, but no acute awareness of it via the senses	Acute awareness of indoor air quality via the senses – typically from sight or smell
Contextual Conditions	Individual level health literacy	Self-reported comfort and skill level accessing written and oral medical information and forms via the BRIEF health literacy assessment	Inadequate health literacy (substantial challenges posed by medical materials, forms, or oral communications)	Adequate health literacy (has skills necessary to access and use health information as well as to exchange information regarding medical content)
	Individual IAQ environmental health knowledge	Participant's lived experience that impacts their comfort accessing, understanding, and using IAQ environmental health information	Little experience with IAQ environmental health information, and little familiarity with topic, stakeholders, and relevant concepts for IAQ	Ample experience with IAQ environmental health information, and familiarity with topic, stakeholders, and relevant concepts for IAQ
	Housing type and proximity to neighbors	Structural aspects & ownership of built home environment	Multifamily or apartment units and/or rental	Single family unit and/or occupant owned

	Existing home behaviors and habits	Habitual or typical home behaviors related to IAQ (incense and candle use, cooking habits, ventilation habits, smoking habits, etc.)	Describes history or habit of actions (or inactions) in the household that reduce indoor air pollution	Describes history or habit within household that may increase indoor air pollution
Action/Inaction Strategies	Purchasing items	Purchased items such as air purifiers or humidifiers to impact home's IAQ	Did not purchase items such as air purifiers or humidifiers for the home	Purchased at least one item such as an air purifier or humidifier for the home
	Home behaviors	Adjusting typical in-home behaviors to impact home's IAQ	Did not change or adjust in-home behaviors to impact the home's IAQ	Adjusted some in-home behaviors to impact the home's IAQ
	Information seeking	Individual's experience looking for information related to indoor air quality or health conditions related to indoor air quality	Has never sought information related to indoor air quality or associated health conditions	Has spent some amount of time seeking information related to indoor air quality or associated health conditions
Intervening Conditions	Perceived agency	Level of confidence in ability to control indoor air quality	Feel defeated in ability to control indoor air quality; do not have access to the resources would like to control indoor air quality; unsure what to do to control indoor air quality	Feel confident in ability to control indoor air quality; feels confident in ability to make changes to address indoor air quality
	Risk perception of IAQ	Level of concern for indoor air quality and potential impact to household members	Have not thought about indoor air quality, or express no concern	Concerned about indoor air quality and potential health impact

	Accessibility of EH information/materials	Clarity and appropriateness of EH materials to facilitate the user's ability to understand information, and to navigate decisions relevant to the information	Encountered EH materials that were received as overly jargon-laden or complex	Encountered EH materials that were received as clear and actionable
Consequences	Perception of access to resources or actions to reduce indoor air pollution	Sense of access to resources or skills/knowledge to achieve healthy IAQ	Feeling discouraged in ability to access resources or act on information to achieve healthy IAQ	Feeling confident in ability to access resources or act on information to achieve healthy IAQ
	Perceived ability to control indoor air quality	Perception of ability to act to maintain or impact own indoor air quality	Describes experiences of attempt to address indoor air quality as unsuccessful with no confidence in future actions being successful	Describes experiences of attempt to address indoor air quality as successful with some degree of hope for future action to be successful
	Sense of security for personal and family health	Degree of concern about family members' health being affected by home's indoor air quality	Has concern about potential impact of air quality on personal or family members' health. Feels concerned about maintaining a healthy home environment	Not concerned about air quality for impact to personal or family members' health. Feels confident in having a 'healthy home' environment

Table S3: Distribution of Baseline Survey Response on Dorchester Air Quality and Interview Sampling Response Rates

	Baseline Survey Response		Qualitative Interviews	
**Perspective of Dorchester Air Quality**	**N**	**Percent**	**Target N for Interviews**	**N Interviewed**
Very Bad	6	8.3	2	5
Bad	12	16.7	3	5
Good	25	34.7	7	1
Very Good	2	2.8	1	2
Never Thought About It	15	20.8	4	5
Uncertain	12	16.7	3	2
**Total**	72	100	20	20

Table S4: Comparison of Demographics for Risk Perception Levels

	Low IAQ Risk Perception (n=7)	High IAQ Risk Perception (n=11)	P-value
**Race**			0.21
American Indian or Alaska Native, Black, Other	0 (0%)	1 (9%)	
Asian	1 (14%)	0 (0%)	
Black or African American	2 (29%)	7 (64%)	
Other	0 (0%)	1 (9%)	
White	4 (57%)	2 (18%)	
**Household Income**			0.12
Less than $20,000	0 (0%)	3 (27%)	
$20,000 to $50,000	1 (14%)	2 (18%)	
$50,000 to $100,000	3 (43%)	5 (45%)	
$100,000 or more	3 (43%)	0 (0%)	
Refused to answer	0 (0%)	1 (9%)	
**Education**			0.12
Less than high school or GED	1 (14%)	0 (0%)	
Highschool diploma or GED	0 (0%)	1 (9%)	
Some college	1 (14%)	2 (18%)	
Bachelor's degree	0 (0%)	5 (45%)	
Postgraduate degree	5 (71%)	3 (27%)	
**BRIEF HL Score (Adequate)**	5 (71%)	6 (55%)	0.83

Table S5: Comparison of Demographics for Perceived Self Agency Levels

	**Negative or Low Perceived Self Agency** (n=5)	**Positive Perceived Self Agency** (n=13)	**P-value**
**Race**			0.6
American Indian or Alaska Native, Black, Other	0 (0%)	1 (8%)	
Asian	0 (0%)	1 (8%)	
Black or African American	4 (80%)	5 (38%)	
Other	0 (0%)	1 (8%)	
White	1 (20%)	5 (38%)	
**Household Income**			0.14
Less than $20,000	2 (40%)	1 (8%)	
$20,000 to $50,000	1 (20%)	2 (15%)	
$50,000 to $100,000	1 (20%)	7 (54%)	
$100,000 or more	0 (0%)	3 (23%)	
Refused to answer	1 (20%)	0 (0%)	
**Education**			0.36
Less than high school or GED	0 (0%)	1 (8%)	
Highschool diploma or GED	1 (20%)	0 (0%)	
Some college	1 (20%)	2 (15%)	
Bachelor's degree	2 (40%)	3 (23%)	
Postgraduate degree	1 (20%)	7 (54%)	
**BRIEF HL Score (Adequate)**	1 (20%)	10 (77%)	0.093
